# Longitudinal differentiation among pelagic populations in a planktic foraminifer

**DOI:** 10.1002/ece3.286

**Published:** 2012-07

**Authors:** Yurika Ujiié, Takahiro Asami, Thibault de Garidel-Thoron, Hui Liu, Yoshiyuki Ishitani, Colomban de Vargas

**Affiliations:** 1 Center for Advanced Marine Core Research, Kochi UniversityKochi 783-8502, Japan; 2 Department of Biology, Shinshu UniversityMatsumoto 390-8621, Japan; 3 CEREGE, CNRS, Aix-Marseille Université, IRD, Technopôle de l’Arbois-MéditerannéeBP80, 13545 Aix-en-Provence Cedex, France; 4 Institute of Marine and Coastal Sciences, Rutgers UniversityNew Brunswick, New Jersey 08901; 5 Japan Agency for Marine-Earth Science and TechnologyYokosuka 237-0061, Japan; 6 UMR CNRS 7144 Evolution du Plancton et PaleOceansStation Biologique, BP74, 29682 Roscoff, France

**Keywords:** Longitudinal gradient, ocean current, phylogeography, planktic foraminifer

## Abstract

Evolutionary processes in marine plankton have been assumed to be dependent on the oceanic circulation system, which transports plankton between populations in marine surface waters. Gene flow facilitated by oceanic currents along longitudinal gradients may efficiently impede genetic differentiation of pelagic populations in the absence of confounding marine environmental effects. However, how responsible oceanic currents are for the geographic distribution and dispersal of plankton is poorly understood. We examined the phylogeography of the planktic foraminifer *Pulleniatina obliquiloculata* in the Indo-Pacific Warm Pool (IPWP) by using partial small subunit ribosomal DNA (SSU rDNA) sequences. We found longitudinal clines in the frequencies of three distinct genetic types in the IPWP area. These frequencies were correlated with environmental factors that are characteristic of three water masses in the IPWP. Noteworthy, populations inhabiting longitudinally distant water masses at the Pacific and Indian sides of the IPWP were genetically different, despite transportation of individuals via oceanic currents. These results demonstrate that populations of pelagic plankton have diverged genetically among different water masses within a single climate zone. Changes of the oceanic circulation system could have impacted the geographic patterns of dispersal and divergence of pelagic plankton.

## Introduction

The phylogeography of pelagic organisms on a global scale is essential for understanding the history of biological responses to oceanic environmental changes. Environmental variables and their geological history govern biogeographic patterns (Slatkin 1987; Glor and Warren 2011). These patterns are associated with the geography of dispersal and gene flow between populations. In the pelagic environment, water masses are separated from one another horizontally by oceanic currents and vertically by the thermocline, which is a sharp decrease of water temperature with depth. Water mass is physically defined by water density, which depends on temperature and salinity. Of these physical properties, temperature plays a major role in structuring global patterns of marine biodiversity (e.g., Snelgrove 2010; Tittensor et al. 2010). Water mass structure can therefore be the primary determinant of the geographic distribution of pelagic organisms.

Oceanic currents also function as conduits between water masses. Accordingly, some studies have demonstrated that oceanic currents disperse marine organisms with high conveyance and determine connectivity between habitats (Treml et al. 2008; White et al. 2010). In particular, western boundary currents, which flow from low to high latitudes along the western margin of oceanic basins, play a role for the latitudinal dispersal of marine plankton (e.g., Barton et al. 2010; Tittensor et al. 2010). However, in spite of such dispersals, pelagic communities are highly differentiated between water masses associated with latitudinal climate zones. These latitudinal gradients potentially confound the effect of many other environmental factors (e.g., sharp environmental gradients such as latitudinal climate differences; Glor and Warren 2011). Further examination of the role of oceanic currents in gene flow between pelagic populations requires a focus on genetic differentiation among populations within a single climate zone.

**Figure 1:**
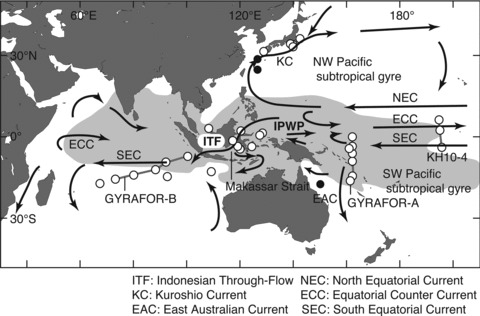
Oceanic setting and sampling locations around the Indo-Pacific Warm Pool (IPWP). The IPWP is highlighted as a hatched area. Each black arrow shows the major path of oceanic current. White circles indicate the present sampling sites and black circles the sites from literature (Darling et al. 2003; Li et al. 2007; Ujiié and Lipps 2009).

Simplistically the oceanic currents in the Indo-Pacific Warm Pool (IPWP) in the tropical climate zone flow in a longitudinal direction. The IPWP is defined as warm surface water that accumulates around the Indonesian Archipelago after being driven across the Pacific Ocean by the westward equatorial current system (Fig. 1). Warm water passes from the western Pacific to the Indian Ocean as the Indonesian Throughflow (ITF). This flux of water is driven by differences in the density of surface water between the southern and northern ends of the Makassar Strait (between the Indonesian islands of Borneo and Sulawesi) (Newton et al. 2011). Most of the heat and salt flux (up to 80%) is accounted for by currents that pass through the upper 300 m of the Makassar Strait after diverging from the Mindanao Current, which is a part of the northwestern Pacific gyre (Gordon and Fine 1996; Schneider 1998; Vranes et al. 2002). The ITF could minimize longitudinal environmental differences in surface water and efficiently transfer marine organisms from the Pacific to Indian sides of the IPWP area. Thus, the IPWP is one of the model areas for determining whether dispersals via oceanic currents have been frequent enough to homogenize genetic structure of marine plankton populations.

Despite this interocean transport system, several studies have shown regional genetic differentiation of marine organisms in the IPWP (e.g., Echinoidea, Malacostraca, and Bivalvia; see Palumbi et al. 1997; Duda and Palumbi 1999; Barber et al. 2000; Benzie et al. 2002; Lind et al. 2007; Ragionieri et al. 2009). However, those studies dealt with neritic organisms that live in coastal habitats including coral reefs. The dispersal of neritic organisms by oceanic currents most probably occurs only during the larval stage. Moreover, the current systems around shallow waters have possibly been changed and even blocked by local geographic changes throughout geologic history (Voris 2000). These complications confound testing the role of oceanic current systems in broad dispersal among populations. In contrast, pelagic holoplankton dwell in pelagic water throughout their life. Phylogeographic studies of pelagic plankton are therefore crucial to examining the role of oceanic current systems in the movement of individuals between distant populations.

Planktic foraminifera, widespread holoplankton, are passively transported by oceanic currents (Bé and Tolderlund 1971; Hemleben et al. 1989). These organisms are unique protists; they are single-celled zooplankton with a calcareous shell. Their morphospecies, identified by shell structure, are distributed in five latitudinal provinces from tropical to polar seas (Bé and Tolderlund 1971; Bé 1977). Moreover, molecular phylogenetic studies have revealed the presence of multigenetic types in a single morphospecies and within their finer scale geographic distributions (compiled in Darling and Wade 2008). They are separated by latitude from polar to temperate zones corresponding to particular hydrographic conditions (de Vargas et al. 1999, 2002; Darling et al. 2004). Intriguingly, many genetic types within warm-water morphospecies show interoceanic distributions (e.g., Darling et al. 1999; de Vargas et al. 1999; Ujiié and Lipps 2009) suggesting that planktic foraminiferal populations have experienced dispersals over long distances.

Recent studies have demonstrated that the distribution of each planktic foraminiferal genetic type is restricted to a small geographic area. *Neogloboquadrina pachyderma* genetic types show narrowly constrained geographic distributions associated with steep temperature gradients in the Antarctic and Arctic circumpolar currents (Darling et al. 2004, 2007). Two genetic types of *Globigerinoides ruber* form two populations in separate narrow basins in the Mediterranean Sea (Aurahs et al. 2009). Moreover, in the subtropical and tropical regions of the Atlantic Ocean, *Globorotalia truncatulinoides* forms two populations, with vertical partitioning according to the structure of adjacent water masses (Ujiié et al. 2010). These populations are distinguished by the opposite coiling phenotypes of their shells, though they maintain gene flow to each other. Thus, planktic foraminifera are potentially excellent tools for assessing the relationship between the oceanic current systems and gene flow in the pelagic ocean.

The partial small subunit ribosomal RNA gene (SSU rDNA) has been the genetic marker used in most cases to classify genetic types of planktic foraminifera (e.g., de Vargas et al. 1997, 1999, 2002; Darling et al. 1999, 2004, 2007; Aurahs et al. 2009; Ujiié and Lipps 2009). Multiple copies of the rRNA gene array exist within single individuals (Darling and Wade 2008). However, no identical copies have been found between the genetic types (Darling and Wade 2008; Aurahs et al. 2009). These genetic types have been identified by two kinds of evidence. First, a clade of each genetic type is robustly supported in a phylogenetic tree (e.g., de Vargas et al. 1997; Ujiié and Lipps 2009). In planktic foraminifera, this divergence indicates no hybridization between different genetic types by means of sexual reproduction (Hemleben et al. 1989). Second, differences among genetic types can clearly be distinguished from intraindividual variation (e.g., Darling et al. 2004, 2007; Ujiié and Lipps 2009; Aurahs et al. 2009). Thus, the partial SSU rDNA is a useful gene marker that can be used to distinguish among populations of planktic foraminifera.

The present study revealed the genetic diversity of the planktic foraminifer *Pulleniatina obliquiloculata* based on a phylogeographic analysis of partial SSU rDNA sequence data. This species is distributed from the tropics to the subtropics, particularly in the IPWP area (Bé 1977). Our surveys of genetic structure along the Indo-Pacific oceanic circulation system showed the presence of clear longitudinal differentiation between pelagic populations in the IPWP.

## Materials and Methods

### Oceanic setting and sample collection

**Figure 2:**
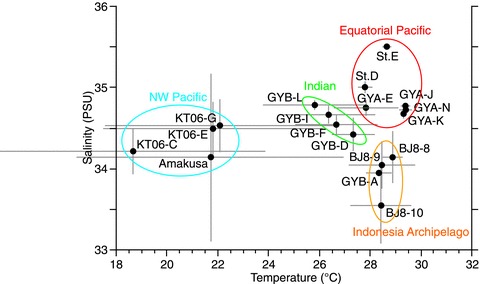
Profile of annual sea surface temperature (SST) and salinity (SSS) at 18 examined sites, where more than 10 specimens were collected. Gray horizontal and vertical bars show the mean values of summer and winter seasons in SST and SSS, respectively. Four water masses defined by T-S profile were circled by four different colors.

In total, we collected 892 specimens of *P. obliquiloculata* at 42 sampling sites during 10 different cruises, most of which took place between June and September from 2006 to 2010 (Table 1). The sampling sites included oceanic biomes ranging from the tropics to temperate regions, particularly at both the Pacific and Indian sides of the IPWP (Table 1; Fig. 1). Sea surface temperature (SST) and sea surface salinity (SSS) patterns at the sampling sites in the Indo-Pacific ocean delimited four main water masses: the Northwest Pacific (Kuroshio area), Indian Ocean, Indonesian Archipelago, and Equatorial Pacific (Fig. 2). Mean SST and SSS at each site were obtained from the World Ocean Atlas 2009 (Locarnini et al. 2010).

**Table 1:** The location of 42 study sites with the number of all examined specimens

Area	Cruise	Site name	Latitude	Longitude	Sp #
Atlantic	CMarZ	CMarZ_0	33°02′N	75°02′W	2
	(April 2006)	CMarZ_1	33°35′N	69°31′W	9
	AMT-8	AMT8_12	27°03′N	21°52′W	1
	(May–June 1999)	AMT8_8	15°09′N	21°02′W	1
		AMT8_3	0°04′N	16°42′W	1
		AMT8_2	4°07'S	15°31′W	1
	AMT-5	St.10	24°135'S	20°99′W	1
	(Sept.–Oct. 1997)	St.14	2°81'S	24°16′W	1
		St.16	0°77′N	25°66′W	1
		St.22	27°41′N	16°42′W	1
NW-	KT06-11	KT06_C	36°23′N	142°57′E	79
Pacific	(June 2006)	KT06_E	34°04′N	140°02′E	71
		KT06_F	34°26′N	139°03′E	4
		KT06_G	33°21′N	140°00′E	31
	Amakusa	Amakusa	32°24′N	129°45′E	18
	(Oct. 2009)				
Indonesian	BJ8-03	BJ8_PT6	2°24′N	120°40′E	1
	(July 2003)	BJ8_PT8	1°43′N	128°46′E	32
		BJ8_PT9	3°55'S	124°51′E	12
		BJ8_PT7	0°58′N	127°48′E	3
		BJ8_PT10	4°51'S	120°08′E	23
		BJ8_PT5	1°36'S	117°31′E	4
		BJ8_PT3	3°53'S	119°23′E	1
SW-	GYRAFOR-A	GYA_E	14°43'S	162°30′E	34
Pacific	(June 2008)	GYA_G	9°00'S	162°32′E	5
		GYA_H	6°07'S	162°31′E	5
		GYA_J	3°52'S	162°32′E	17
		GYA_K	0°00	162°32′E	14
		GYA_N	4°30'S	161°12′E	24
		GYA_S	16°15'S	162°53′E	6
		GYA_T	17°56'S	162°41′E	1
Central	KH10-4	St. C	7°09′N	166°33′W	2
Pacific	(Sept. 2010)	St. D	2°42′N	165°33′W	64
		St. E	5°40'S	164°13′W	144
Indian	GYRAFOR-B	GYB_6	3°13′N	108°28′E	1
		GYB_A	7°22'S	100°40′E	11
	(July–Aug. 2007)	GYB_C	9°30'S	92°25′E	3
		GYB_D	12°07'S	88°32′E	103
		GYB_F	14°12'S	80°13′E	58
		GYB_I	15°57'S	73°16′E	51
		GYB_L	17°21'S	67°38′E	48
	Melville	St. 19	15°45'S	86°46′E	1
	(May–June 2003)	St. 26	14°29'S	113.27′E	3
				TOTAL	892

Samples were obtained by a plankton-net system with 63- or 100-μm mesh except for the samples taken from the Sargasso Sea, where we used a 355-μm mesh-size net. In the Pacific and Indian Oceans, we carried out depth-specific samplings in five to nine layers from the surface to a depth of 400 m at each site. In the Southwest (SW) Pacific and Indian Oceans, we used the CEREGE MultiNet Midi (HydroBios, Kiel, Germany), which was equipped with an opening–closing 100-μm mesh net. In the Northwest (NW) and Central Pacific, the Motoda (MTD) closing net system (Rigo, Tokyo, Japan) with 63-μm mesh was towed horizontally. We obtained 775 specimens with these systems. We obtained the other 117 specimens from samples collected with vertical tows in the upper 150–200 m of the water column at each location.

We isolated and cleaned each specimen in filtered seawater under a microscope. Each individual specimen was separately preserved with the GITC* (guanidine-base) buffer at –20°C in an onboard freezer. Genomic DNA was extracted by the GITC* protocol (Ujiié et al. 2010).

### DNA amplification

PCR amplifications of the terminal 3′ end of the SSU rDNA was performed as described by de Vargas et al. (1997). Approximately 1070 base pairs (bps) were amplified using GoTaq Flexi DNA Polymerase (Promega, Madison, WI) with the foraminifera-specific primer S14F1 (5′-AAG GGC ACC ACA AGA ACG C-3′) coupled with the universal primer SBf (5′-TGA TCC ATC (AG)GC AGG TTC ACC TAC-3′) (de Vargas et al. 1997). The amplification consisted of 35 cycles at 95°C for 30 sec, 56°C for 30 sec, 72°C for 1.5 min, with final extending at 72°C for 10 min. We randomly selected at least 10 specimens from each sampling area (in total, 66 individuals) for sequencing. We cloned these PCR products with a TOPO TA Cloning Kit (Invitrogen, Carlsbad, CA). We sequenced from four to eight clones in forward and reverse directions using the primers M13F (5′-GTA AAA CGA CGG CCA G-3′) and M13R (5′-CAG GAA ACA GCT ATG AC-3′) with an ABI prism 3100 sequencer (Applied Biosystems, Foster, CA) at the Station Biologique (Roscoff, France) and an ABI prism 3130 sequencer (Applied Biosystems) at Shinshu University (Matsumoto, Japan). We confirmed that all clones from an individual showed commonality to each other. All cloned sequences from 66 individuals were deposited in Genbank with accession numbers AB636683–AB636926.

### Phylogenetic analysis

We manually aligned 66 individual sequences with seven published sequences of *P. obliquiloculata* (Darling et al. 2003; Li et al. 2007; Ujiié and Lipps 2009) using the MacGDE software (Linton 2005). In total, 1014 unambiguously aligned nucleotide sites were used for the phylogenetic analysis. We selected the general time reversible model (GTR; Yang 1994a) with the gamma distribution approximated by eight discrete categories (gamma; Yang 1994b) as the best-fit model of nucleotide substitution for this dataset. We used MrModelTest v. 2.2 (Nylander 2004) to select the best-fit model. We conducted Bayesian phylogenetic analyses with MrBayes 3.1.2 (Ronquist and Huelsenbeck 2003) under the optimal model. The Markov chain Monte Carlo (MCMC) process was set so that four chains (three heated and one cold) ran simultaneously. Two independent runs were conducted for one million generations after reaching stationaries. We sampled trees and log-likelihood values at 100-generation intervals. We confirmed that the estimated parameters agreed with each other between the two independent runs. We then pooled all trees after the burn-in period (10,000 generations). We estimated posterior probabilities on the basis of the pooled trees. Maximum-likelihood analyses were performed with Treefinder (Jobb 2008) by using the same model of evolution as noted earlier. Bootstrap support was based on 1000 replicates.

### Identification of genetic types

Based on the phylogeny mentioned above, we identified three individual genetic types that clustered in three groups. We estimated the mean sequence divergence between genetic types by pairwise sequence comparisons among 73 sequences following a maximum-likelihood model in PAUP* 4.0b10 (Swofford 2002).

We developed two steps to rapidly identify the genetic type of 892 examined specimens. First, we applied a PCR product to restriction fragment length polymorphism (RFLP) analysis by using the enzyme PsiI (New England Biolabs, Beverly, MA), which cuts at the palindromic sequence TTA/TAA. We detected two distinct patterns for types I + IIa and IIb after migration on 1% agarose gel. Second, we directly sequenced both first and last variable regions (∼200 bp) in the 3′ end of SSU rDNA of 747 specimens to classify types I and IIa. We amplified the first variable region with the primers S14F1 and S15r (5′-GAA CTA AGA ACG GCC ATG CAA-3′) and the last one with the primers S19rf (5′-CTA GGA ATG CCT (CT)GT ACG GG-3′) and SBf.

### Statistical analyses

We used Spearman's rank correlation analysis to test the relationships of genetic-type frequency with geographic (longitude and latitude) and environmental (SST and SSS) factors. We used sampling sites where at least 10 individuals were collected to reduce sampling errors in frequency estimation. We confirmed that the results of these tests did not change in terms of statistical significance even when we used sites where at least 20 individuals were collected.

## Results

### Genetic types of *P. obliquiloculata*

**Figure 3:**
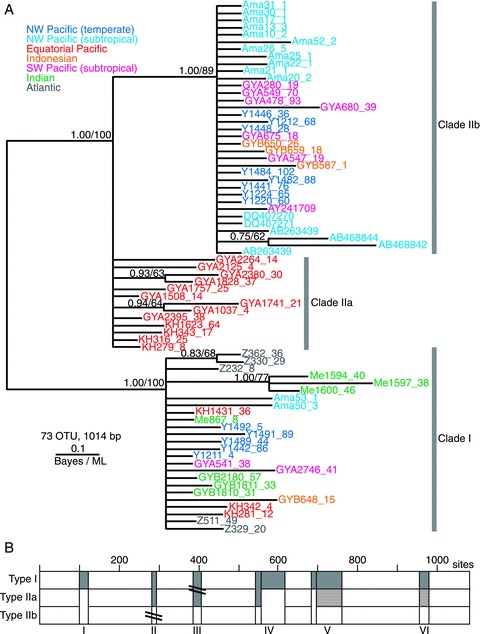
(A) Phylogenetic reconstruction (Bayesian analysis, 50% majority consensus tree) based on the SSU rDNA sequences from 73 *P. obliquiloculata* specimens. The sampling areas are shown in seven different colors. Numbers at each node are posterior probabilities and bootstrap values. (B) Diagram of the alignment of three genetic types. Hatched ranges (variable regions I–VI) show specific sites to identify the genetic types. Transverse lines indicate the place cutoff by the restriction enzyme for the restriction fragment length polymorphism (RFLP) analysis.

Our SSU rDNA phylogeny detected the presence of two clades (clades I and II) supported with 1.00 posterior probability (PP) and a 100% bootstrap value (BV) (Fig. 3A). Clade II was divided into two subclades (named IIa and IIb). Clade IIa was paraphyletic with clade IIb but was clearly separated from the monophyletic clade IIb on the basis of 1.00 PP and 89% BV. On average, bp sequences differed by 1.2%, 1.5%, and 0.5% between clades I and IIa, I and IIb, and IIa and IIb, respectively.

Foraminiferal SSU rDNA sequences have shown relatively high diversity among eukaryotes due to their rapid evolution and long-fragment insertions (Pawlowski et al. 1996; Ertan et al. 2004; Schweizer et al. 2008). In the 3′ end of SSU rDNA, six variable regions have been especially recognized as regions I–VI by de Vargas et al. (1997). Three of them (regions III, IV, and VI) are commonly observed from diverse eukaryotic groups; however, the others (regions I, II, and V) have been found specifically in foraminifera as insertion parts (Ertan et al. 2004; Schweizer et al. 2008). In most cases, we found the sequence variations in these six regions attributable to distinct clades of the SSU rDNA phylogeny. The sequences of clades I and IIb were identical in all six regions (Figs. 3B and S1). Clade IIa differed from the other two clades at two variable regions V and VI, and shared the same sequences with clades I or IIb at the other regions. Even though intraindividual copies differed in a few bps, they had the same sequences in the six variable regions that characterized each clade. Based on their distinct genetic differences confirmed by the phylogenetic analysis and sequence alignment, we defined three genetic types I, IIa, and IIb, which correspond to clades I, IIa, and IIb, respectively. We classified all previously published specimens from the East China Sea and Great Barrier Reef into type IIb (Darling et al. 2003; Li et al. 2007; Ujiié and Lipps 2009).

Because the restriction enzyme PsiI that we used for the RFLP method cut the examined sequences in the variable regions II and III, the RFLP patterns distinguished types I + IIa and IIb (Fig. 3B). Direct-sequencing analyses divided types I and IIa by identical sequences of the variable regions I and VI. By means of the two analytical steps, we classified all of the specimens into three genetic types: 536 specimens as type I, 211 specimens as type IIa, and 145 specimens as type IIb (Table 2).

**Table 2:** The number of genetic types at 42 study sites and depth-different layers

St. name	Depth(m)	I	IIa	IIb	Total	St. name	Depth(m)	I	IIa	IIb	Total
ATLANTIC						BJ8_PT8		9	0	23	32
CMarZ_0		2	0	0	2	BJ8_PT9		10	0	2	12
CMarZ_1	Site total	9	0	0	9	BJ8_PT7		1	0	2	3
	0–25	2	0	0	2	BJ8_PT10		17	0	6	23
	25–50	4	0	0	4	BJ8_PT5		1	0	3	4
	50–100	2	0	0	2	BJ8_PT3		1	0	0	1
	100–150	1	0	0	1	SW PACIFIC					
AMT8_12		1	0	0	1	GYA_E	Site total	5	2	27	34
AMT8_8		0	0	1	1		0–20	2	1	8	11
AMT8_3		1	0	0	1		35–50	0	0	1	1
AMT8_2		1	0	0	1		50–70	0	0	5	5
St.10		1	0	0	1		70–90	2	1	7	10
St.14		1	0	0	1		100–125	1	0	2	3
St.16		1	0	0	1		125–150	0	0	2	2
St.22		1	0	0	1		150–200	0	0	1	1
NW PACIFIC							200–400	0	0	1	1
KT06_C	0	62	0	17	79	GYA_G	Site total	3	0	2	5
KT06_E	Site total	50	0	21	71		60–80	1	0	0	1
	0	10	0	3	13		120–180	2	0	0	2
	20	12	0	3	15	GYA_H	Site total	0	0	6	6
	75	4	0	4	8		60–80	0	0	1	1
	100	3	0	4	7		80–100	0	0	2	2
	120	8	0	4	12		100–120	0	0	1	1
	335	7	0	2	9		120–140	0	0	1	1
	350	3	0	0	3		125–160	0	0	1	1
KT06_F	50	3	0	1	4	GYA_J	Site total	0	13	2	15
KT06_G	50	27	0	4	31		0–30	0	4	0	4
Amakusa		2	0	16	18		30–60	0	5	1	6
INDONESIAN							60–90	0	2	1	3
BJ8_PT6		1	0	0	1		90–120	1	2	0	2
GYA_K	Site total	1	11	2	14		58–85	6	0	3	9
	0–30	0	4	2	6	GYB_C	Site total	3	0	0	3
	30–60	1	2	0	3		0–30	2	0	0	2
	90–120	0	3	0	3		30–62	1	0	0	1
	150–250	0	2	0	2	GYB_D	Site total	103	0	0	103
GYA_N	Site total	3	20	1	24		0–53	40	0	0	40
	30–60	1	7	0	8		53–70	48	0	0	48
	60–85	1	4	1	6		70–90	15	0	0	15
	85–120	0	3	0	3	GYB_F	Site total	58	0	0	58
	120–160	1	5	0	6		0–36	14	0	0	1
	160–225	0	1	0	1		36–55	10	0	0	10
GYA_S	42.5	1	1	4	6		55–88	2	0	0	2
GYA_T	50	1	0	0	1		88–130	2	0	0	2
CENTRAL PACIFIC							130–165	2	0	0	2
St. C	65	0	2	0	2		160–190	4	0	0	4
St. D	Site total	26	38	0	64		190–250	24	0	0	24
	30	4	5	0	9	GYB_I	Site total	51	0	0	51
	90	3	6	0	9		0–30	24	0	0	24
	120	4	3	0	7		36–55	12	0	0	12
	180	5	0	0	5		80–106	5	0	0	5
	250	5	9	0	14		106–139	4	0	0	4
	400	5	15	0	20		139–160	2	0	0	2
St. E	Site total	20	124	0	144		160–225	1	0	0	1
	30	9	31	0	40		225–303	3	0	0	3
	70	1	17	0	18	GYB_L	0–100	48	0	0	48
	100	5	21	0	26	St. 19		1	0	0	1
	150	4	41	0	45	St. 26		3	0	0	3
	250	1	14	0	15	TOTAL		536	211	145	892
INDIAN OCEAN											
GYB_6		0	0	1	1						
GYB_A	Site total	7	0	4	11						
	0–58	1	0	1	2						

### Vertical distribution of genetic types

**Figure 4:**
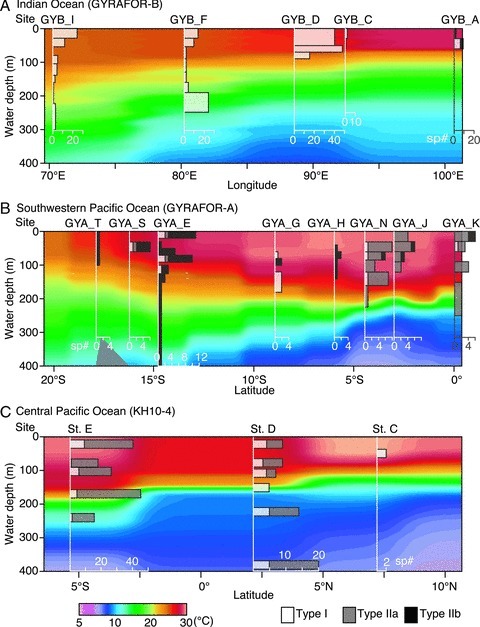
Vertical distribution of three genetic types at each sampling site. Background is a profile of water temperature. (A) Site of the GYRAFOR-B cruise in the Indian Ocean, (B) sites of the GYRAFOR-A cruise in the southwest Pacific Ocean, and (C) sites of the KH10–4 cruise in the central Pacific Ocean.

Depth-specific samplings revealed the vertical distributions of the three genetic types in the Indian, and SW and central Equatorial Pacific oceans (Fig. 4). Most specimens were found in the upper 150 m of the water column in all collections. This depth corresponds closely to the annual thermocline. At the SW and Central Pacific sites, two or three genetic types occurred frequently together in the same layer.

### Geographic distribution of genetic types

**Figure 5:**
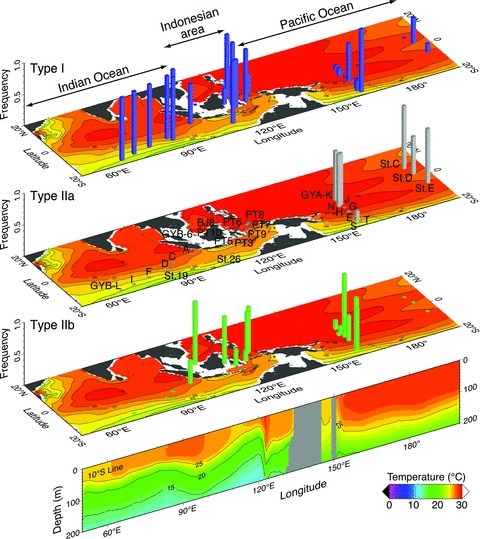
The counter line indicates sea surface temperature. The bottom picture shows the vertical temperature profile along the latitude 10°S. Mean hydrographic values in August from 2006 to 2010, obtained from the Global Ocean Data Assimilation System (http://www.esrl.noaa.gov/psd), were used.

We found type I specimens in all of the areas we studied. In contrast, type IIa was exclusively found in the equatorial Pacific area, and type IIb mainly in the Pacific including the Indonesian Archipelago (Table 2; Fig. 5). All three types coexisted in the equatorial area of the western Pacific (Fig. 5). Several geographic patterns were evident: types I and IIb were found in the NW Pacific, whereas types I and IIa were found in the Equatorial Pacific. Type I alone was abundant in the Atlantic and Indian Oceans, except for two sampling sites. One specimen of type IIb was detected at a single site in the Atlantic. Type IIb specimens were also found at a site in the Indian Ocean located at the boundary between the Indian and Pacific Oceans around the island of Java.

**Figure 6:**
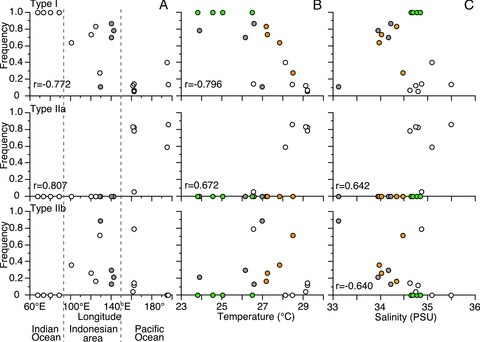
(A) Frequencies of genetic types plotted against longitude. Significant correlation coefficients (*P* < 0.05) are shown in each graph. Gray-colored symbols show the data from four sites in the temperate area on the northwestern Pacific. (B) Frequencies of genetic types plotted against SSTs. Each colored symbol shows the data from the sites in the Indian Ocean (green), NW Pacific Ocean (gray), SW Pacific Ocean (white), and Indonesian area (orange). (C) Frequencies of genetic types plotted against SSSs. Each colored symbol is same as that of Figure 6B.

In the Indo-Pacific Ocean, the frequency of each genetic type at each sampling site was significantly correlated with longitude (Fig. 6A). The frequency of type I was negatively correlated with longitude (*r* = –0.772, *P* < 0.001), whereas those of type IIa displayed a positive correlation with longitude (*r* = 0.807, *P* < 0.001). Only type IIb did not show a significant correlation with longitude. In contrast, the relationship between the frequencies of three types and latitude was not significant.

### Correlation between genetic type and environmental factors

The frequencies of types I and IIa were correlated with SST. Type I was negatively correlated with SST (*r* = –0.796, *P* < 0.001) (Fig. 6B). In contrast, type IIa was positively correlated with SST (*r* = 0.672, *P* < 0.01). The frequencies of types IIa and IIb were significantly correlated with SSS (Fig. 6C). Type IIb was negatively correlated with SSS (*r* = –0.640, *P* < 0.01), whereas type IIa showed a positive correlation (*r* = 0.642, *P* < 0.01).

## Discussion

### Environmental factors associated with geographic clines in genetic-type frequency

We found three distinct genetic types in the single morpho-species *P. obliquiloculata*. At each sampling site, two or three genetic types were distributed vertically at the same depth in the water column (Fig. 4). We confirmed that there was no depth partitioning among the three genetic types. However, the frequency of each genetic type varied among the sampling sites within a narrow latitudinal range of the Indo-Pacific Ocean (Fig. 6A). In the Indonesian area, the frequency of type I decreased toward the Pacific side, where type IIb was abundant. At the sites closer to the Equatorial Pacific, type IIa was most predominant. The spatial distributions of the three genetic types thus showed clear longitudinal gradients among the Indian Ocean, Indonesian Archipelago, and Pacific Ocean.

The frequency of each genetic type was correlated with the SST and SSS in the observed area (Fig. 6B and C). Type IIa was relatively frequent where SST (>26°C) and SSS were high. Type IIb was frequent where SSS was low. These environmental conditions match the hydrographic properties of three water masses in the IPWP: Indian Ocean, Indonesian Archipelago, and Equatorial Pacific (Fig. 2). Even within this narrow latitudinal zone, these water masses are delimited by SST and SSS. These indicate that three populations are genetically differentiated among the water masses.

Most of the genetic types detected among the planktic foraminifera show latitudinal separation into geographic distributions (compiled in Darling and Wade 2008; Morard et al. 2011). These geographic distributions are correlated with hydrographic properties (e.g., SST, SSS), which change with latitude according to the structure of the water masses along the ocean province (e.g., de Vargas et al. 2001, 2002; Morard et al. 2011). In the high-latitude zone, the geographic distributions of *N. pachyderma* genetic types are separated on the basis of hydrographic partitioning associated with strong temperature gradients (Darling et al. 2007). Moreover, at a finer scale, opposite coiling phenotypes in a single genetic type of *G. truncatulinoides* are separately distributed between adjacent water masses (Ujiié et al. 2010). These populations were vertically segregated along the boundary of different water masses between the sea surface and a depth of 400 m. These studies suggest that populations of planktic foraminiferal genetic types are established in water masses. Likewise, the present study demonstrates that longitudinal separation of populations is related to water mass structure.

### Function of oceanic current

The present results on spatial distribution of planktic foraminiferal genetic types provide new insights into their dispersal abilities at the population level. The most striking result is that populations of *P. obliquiloculata* were genetically differentiated in the IPWP areas along a longitudinal transect. We therefore address in the following paragraphs two functions of oceanic currents: (1) separation of water masses, each of which is inhabited by a population, and (2) transport of individuals among water masses.

No type IIa specimens were obtained from the Indian Ocean, in spite of our thorough surveys across the IPWP (Fig. 5). The geographic distribution of type IIa was restricted to the SW Pacific gyre bordered by the South Equatorial Current (SEC) and East Australian Current (EAC), and under the Equatorial Countercurrent (ECC) which is diverged from the SEC (Figs. 1 and 5). Contrarily, this type was not found in the NW Pacific gyre, which is another oceanic current system in the Pacific. In the IPWP oceanic current system, the ITF advects water from the NW Pacific to the Indian Ocean side of the IPWP (Gordon and Fine 1996; Schneider 1998; Vranes et al. 2002). Type IIa individuals would not be carried to the Indian Ocean side by this westward-flowing current. Thus, the current system could prevent the dispersal of type IIa specimens from the SW Pacific to other areas, and demonstrates that an oceanic current can function not only as a separator of water masses but also as a hydrographic barrier against dispersal of pelagic plankton.

The longitudinal distribution pattern of type IIb across the Indonesian Archipelago suggests that this type is dispersed to the Indian Ocean through the ITF. We detected type IIb only at the easternmost site on the Indian Ocean side of the IPWP, though all specimens were identified as type I further west in the Indian Ocean. Our surveys were performed during the Asian summer monsoon that provides strong westerly flow from the Pacific to Indian Oceans via the ITF (Potemra 1999; Newton et al. 2011). Despite the interoceanic flow, populations were differentiated in the frequencies of genetic types between the Indian Ocean and Pacific sides of the IPWP. In another study, two different populations of *G. ruber* were distributed at each of two basins that are adjacently located under the same current in the Mediterranean Sea (Aurahs et al. 2009). Two of five *G. ruber* genetic types were found only in either one of the two populations, which were attributed to niche partitioning. However, this interpretation remains questionable without knowing their ecological or physiological characteristics that may allow differential adaptation to environmental conditions to be examined. Although the frequency of *P. obliquiloculata* type IIb was here correlated with SSS, there is no sufficient evidence to argue for evolutionary adaptation of the present populations to different salinities or other environmental conditions.

The present study examined the hypothesis that gene flow among populations is enhanced by passive transport through oceanic currents (Bé and Tolderlund 1971; Snelgrove 2010; Tittensor et al. 2010). We showed robust evidence of longitudinal differentiation between adjacent populations in the absence of any physical barriers. This result indicates that gene flow by passive transport between populations is limited in effect against the hypothesis. Despite the through-flow of water by oceanic currents, the geographic patterns of water masses have been controlled by changes in the oceanic circulation system during the geologic history. These changes could have impacted the geographic patterns of dispersal and divergence in pelagic plankton.

## Supporting Information

**Figure S1.** Alignment of partial SSU rDNA sequeuce, which represents each of three genetic types.
