# Supplementary material for: Longitudinal differentiation among pelagic populations in a planktic foraminifer
Source: Ecol Evol. 2012 Jul;2(7):1725–37. doi: 10.1002/ece3.286 (PMC3434911; doi:10.1002/ece3.286)
Supplement: Supplementary file 1 [file ece30002-1725-SD1.pdf]

Fig. S1

```
                     1                                                            60
Type I   GYB2180_57  GCACCACAAG AACGCGTGGA GCATGTGGCT TAATTTGACT CAACGCGGGA AATCTTACCG
         Y1211_4     GCACCACAAG AACGCGTGGA GCATGTGGCT TAATTTGACT CAACGCGGGA AATCTTACCG
Type IIa KH316_25    GCACCACAAG AACGCGTGGA GCATGTGGCT TAATTTGACT CAACGCGGGA AATCTTACCG
         GYA2395_38  GCACCACAAG AACGCGTGGA GCATGTGGCT TAATTTGACT CAACGCGGGA AATCTTACCG
Type IIb Y1220_60    GCACCACAAG AACGCGTGGA GCATGTGGCT TAATTTGACT CAACGCGGGA AATCTTACCG
         GYB650_26   GCACCACAAG AACGCGTGGA GCATGTGGCT TAATTTGACT CAACGCGGGA AATCTTACCG

                     61                                                          120
Type I   GYB2180_57  GGTCCGGACA CACTGAGGAT TGACAGGCAA TATCTATTAA AAGATTATAA ATATTCTTTT
         Y1211_4     GGTCCGGACA CACTGAGGAT TGACAGGCAA TATCTATTAA AAGATTATAA ATATTCTTTT
Type IIa KH316_25    GGTCCGGACA CACTGAGGAT TGACAGGCAA TATCTATTAA AAGATTTAAA A----CTCTT
         GYA2395_38  GGTCCGGACA CACTGAGGAT TGACAGGCAA TATCTATTAA AAGATTTAAA A----CTCTT
Type IIb Y1220_60    GGTCCGGACA CACTGAGGAT TGACAGGCAA TATCTATTAA AAGATTTAAA A----CTCTT
         GYB650_26   GGTCCGGACA CACTGAGGAT TGACAGGCAA TATCTATTAA AAGATTTAAA A----CTCTT

                     121                                                         180
Type I   GYB2180_57  AATAGTGTTA AATATGCTAG TCCTTTCATG ATTATGTGAT AGGTGGTGCA TGGCCGTTCT
         Y1211_4     AATAGTGTTA AATATGCTAG TCCTTTCATG ATTATGTGAT AGGTGGTGCA TGGCCGTTCT
Type IIa KH316_25    AATAGTGTTA AATATGCTAG TCCTTTCATG ATTATGTGAT AGGTGGTGCA TGGCCGTTCT
         GYA2395_38  AATAGTGTTA AATATGCTAG TCCTTTCATG ATTATGTGAT AGGTGGTGCA TGGCCGTTCT
Type IIb Y1220_60    AATAGTGTTA AATATGCTAG TCCTTTCATG ATTATGTGAT AGGTGGTGCA TGGCCGTTCT
         GYB650_26   AATAGTGTTA AATATGCTAG TCCTTTCATG ATTATGTGAT AGGTGGTGCA TGGCCGTTCT

                     181                                                         240
Type I   GYB2180_57  TAGTTCGTGG AGTGATCTGT CTGCTTAATT GCGTTTCACT AAGGGCCCAT AAATTCAAGG
         Y1211_4     TAGTTCGTGG AGTGATCTGT CTGCTTAATT GCGTTTCACT AAGGGCCCAT AAATTCAAGG
Type IIa KH316_25    TAGTTCGTGG AGTGATCTGT CTGCTTAATT GCGTTTCACT AAGGGCCCAT AAATTCAAGG
         GYA2395_38  TAGTTCGTGG AGTGATCTGT CTGCTTAATT GCGTTTCACT AAGGGCCCAT AAATTCAAGG
Type IIb Y1220_60    TAGTTCGTGG AGTGATCTGT CTGCTTAATT GCGTTTCACT AAGGGCCCAT AAATTCAAGG
         GYB650_26   TAGTTCGTGG AGTGATCTGT CTGCTTAATT GCGTTTCACT AAGGGCCCAT AAATTCAAGG

                     241                                                         300
Type I   GYB2180_57  TATGTTAGCT ATTGTTTCCC TATTGACCCC TTATTTAATA AGCGCGTGTC TTTATGGGTT
         Y1211_4     TATGTTAGCT ATTGTTTCCC TATTGACCCC TTATTTAATA AGCGCGTGTC TTTATGGGTT
Type IIa KH316_25    TATGTTAGCT ATTGTTTCCC TATTGACCCC TTATTTAATA AGCGCGTGTC TTTATGGGTT
         GYA2395_38  TATGTTAGCT ATTGTTTCCC TATTGACCCC TTATTTAATA AGCGCGTGTC TTTATGGGTT
Type IIb Y1220_60    TATGTTAGCT ATTGTTTCCC TATTGACCCC TTATCTTATA AGCGCGTGTC TTTATGGGTT
         GYB650_26   TATGTTAGCT ATTGTTTCCC TATTGACCCC TTATCTTATA AGCGCGTGTC TTTATGGGTT

                     301                                                         360
Type I   GYB2180_57  AAACATTGCG CATGCTGTTG GGTCCTGAAA GCAACGAACG TGACCGCAAC GTCTTGTTGC
         Y1211_4     AAACATTGCG CATGCTGTTG GGTCCTGAAA GCAACGAACG TGACCGCAAC GTCTTGTTGC
Type IIa KH316_25    AAACATTGCG CATGCTGTTG GGTCCTGAAA GCAACGAACG TGACCGCAAC GTCTTGTTGC
         GYA2395_38  AAACATTGCG CATGCTGTTG GGTCCTGAAA GCAACGAACG TGACCGCAAC GTCTTGTTGC
Type IIb Y1220_60    AAACATTGCG CATGCTGTTG GGTCCTGAAA GCAACGAACG TGACCGCAAC GTCTTGTTGC
         GYB650_26   AAACATTGCG CATGCTGTTG GGTCCTGAAA GCAACGAACG TGACCGCAAC GTCTTGTTGC

                     361                                                         420
Type I   GYB2180_57  CTTTATCTTG TTATATCTAT TAAATAGAAT AACTAACAGA GGCTAATCTA AAACTAGACG
         Y1211_4     CTTTATCTTG TTATATCTAT TAAATAGAAT AACTAACAGA GGCTAATCTA AAACTAGACG
Type IIa KH316_25    CTTTATCTTG TTATATCTAT TAAATAGAAT AACTAACAGA GGCTAATCTA AAACTAGACG
         GYA2395_38  CTTTATCTTG TTATATCTAT TAAATAGAAT AACTAACAGA GGCTAATCTA AAACTAGACG
Type IIb Y1220_60    CTTTATCTTG TTATATCTAT TAAATAGAAT AACTAACAGA GGCTAATCTA AAACTAGACG
         GYB650_26   CTTTATCTTG TTATATCTAT TAAATAGAAT AACTAACAGA GGCTAATCTA AAACTAGACG
```

		421					480
Type I	GYB2180_57	GACCGCTGTT	TCTTTCTTAA	ACCAGAGGAA	GGTTGCGGCA	ATAACAGGTC	TGTGATGCCC
	Y1211_4	GACCGCTGTT	TCTTTCTTAA	ACCAGAGGAA	GGTTGCGGCA	ATAACAGGTC	TGTGATGCCC
Type IIa	KH316_25	GACCGCTGTT	TCTTTCTTAA	ACCAGAGGAA	GGTTGCGGCA	ATAACAGGTC	TGTGATGCCC
	GYA2395_38	GACCGCTGTT	TCTTTCTTAA	ACCAGAGGAA	GGTTGCGGCA	ATAACAGGTC	TGTGATGCCC
Type IIb	Y1220_60	GACCGCTGTT	TCTTTCTTAA	ACCAGAGGAA	GGTTGCGGCA	ATAACAGGTC	TGTGATGCCC
	GYB650_26	GACCGCTGTT	TCTTTCTTAA	ACCAGAGGAA	GGTTGCGGCA	ATAACAGGTC	TGTGATGCCC

		481					540
Type I	GYB2180_57	TTAGATGTTC	CGGGCTGCAC	ACGTGCTACA	ATGATCAGTA	CAGTGAGCAT	CTCAATTTTA
	Y1211_4	TTAGATGTTC	CGGGCTGCAC	ACGTGCTACA	ATGATCAGTA	CAGTGAGCAT	CTCAATTTTA
Type IIa	KH316_25	TTAGATGTTC	CGGGCTGCAC	ACGTGCTACA	ATGATCAGTA	CAGTGAGCAT	CTCAATTTTA
	GYA2395_38	TTAGATGTTC	CGGGCTGCAC	ACGTGCTACA	ATGATCAGTA	CAGTGAGCAT	CTCAATTTTA
Type IIb	Y1220_60	TTAGATGTTC	CGGGCTGCAC	ACGTGCTACA	ATGATCAGTA	CAGTGAGCAT	CTCAATTATA
	GYB650_26	TTAGATGTTC	CGGGCTGCAC	ACGTGCTACA	ATGATCAGTA	CAGTGAGCAT	CTCAATTATA

		541					600
Type I	GYB2180_57	TACACCGTAT	TTAGCGCTTA	GATGCGATTA	TTGGCTCTTT	TAGTGTCTTT	TAATTGTATT
	Y1211_4	TACACCGTAT	TTAGCGCTTA	GATGCGATTA	TTGGCTCTTT	TAGGGTCTTT	TAATTGTATT
Type IIa	KH316_25	TACACCGTAT	TTAGCGCTAA	GATATGATTA	TTGGCTCTTT	TAGGGTCTTT	TAATTGTATT
	GYA2395_38	TACACCGTAT	TTAGCGCTAA	GATATGATTA	TTGGCTCTTT	TAGAGTCTTT	TAATTGTATT
Type IIb	Y1220_60	TACACCGTAT	TTAGCGCTAA	GATATGATTA	TTGGCTCTTT	TAGGGTCTTT	TAATTGTATT
	GYB650_26	TACACCGTAT	TTAGCGCTAA	GATATGATTA	TTGGCTCTTT	TAGGGTCTTT	TAATTGTATT

		601					660
Type I	GYB2180_57	TCTAATGCGC	GCGGTAAAGC	CTGCTTCGAG	AGTAAGTGGG	TAATCCATTG	GAAGTAATGA
	Y1211_4	TCTAATGCGC	GCGGTAAAGC	CTGCTTCGAG	AGTAAGTGGG	TAATCCATTG	GAAGTAATGA
Type IIa	KH316_25	TCTTATGCGC	GCGGTAAAGC	CTGCTTCGAG	AGTAAGTGGG	TAATCCATTG	GAAGTAATGA
	GYA2395_38	TCTTATGCGC	GCGGTAAAGC	CTGCTTCGAG	AGTAAGTGGG	TAATCCATTG	GAAGTAATGA
Type IIb	Y1220_60	TCTTATGCGC	GCGGTAAAGC	CTGCTTCGAG	AGTAAGTGGG	TAATCCATTG	GAAGTAATGA
	GYB650_26	TCTTATGCGC	GCGGTAAAGC	CTGCTTCGAG	AGTAAGTGGG	TAATCCATTG	GAAGTAATGA

		661					720
Type I	GYB2180_57	TTTCTCTTTA	TTATAGCACA	CCTATATACG	GCATTCATTC	CCGGGATGAC	TAGTTTCGTC
	Y1211_4	TTTCTCTTTA	TTATAGCACA	CCTATATACG	GCATTCATTC	CCGGGATGAC	TAGTTTCGTC
Type IIa	KH316_25	TTTCTCTTTA	T-ATAGCACA	CCTATATACG	GCATTCATTC	CCGGGATGAC	TAGTTTCGTC
	GYA2395_38	TTTCTCTTTA	T-ATAGCACA	CCTATATACG	GCATTCATTC	CCGGGATGAC	TAGTTTCGTC
Type IIb	Y1220_60	TTTCTCTTTA	T-ATAGCACA	CCTATATACG	GCATTCATTC	CCGGGATGAC	TAGTTTCGTC
	GYB650_26	TTTCTCTTTA	T-ATAGCACA	CCTATATACG	GCATTCATTC	CCGGGATGAC	TAGTTTCGTC

		721					780
Type I	GYB2180_57	TTTTTTGTGC	GAATGTAATG	TATT-CTTTA	TCCGTATGTG	CGATTGTCAA	TTCATGGTGG
	Y1211_4	TTTTTTGTGC	GAATGTAATG	TATT-CTTTA	TCCGTATGTG	CGATTGTCAA	TTCATGGTGG
Type IIa	KH316_25	TTTTTTGTGC	GAATGTAATG	TATT-CTT-A	TCCGTATGTG	CGATTGTCAA	TTCATGGTGG
	GYA2395_38	TTTTTTGTGC	GAATGTAATG	TATT-CTT-A	TCCGTATGTG	CGATTGTCAA	TTCATGGTGG
Type IIb	Y1220_60	TTTTTTGTGC	GAATGTAATG	TATTTCTT-A	TCCGTATGTG	CGATTGTCAA	TTCATGGTGG
	GYB650_26	TTTTTTGTGC	GAATGTAATG	TATTTCTT-A	TCCGTATGTG	CGATTGTCAA	TTCATGGTGG

		781					840
Type I	GYB2180_57	GGACAGACCA	TTGTTAATTG	TTGGTCTCGG	TCTTAACTAG	GAATGCCTTG	TACGGGTCTT
	Y1211_4	GGACAGACCA	TTGTTAATTG	TTGGTCTCGG	TCTTAACTAG	GAATGCCTTG	TACGGGTCTT
Type IIa	KH316_25	GGACAGACCA	TTGTTAATTG	TTGGTCTCGG	TCTTAACTAG	GAATGCCTTG	TACGGGTCTT
	GYA2395_38	GGACAGACCA	TTGTTAATTG	TTGGTCTCGG	TCTTAACTAG	GAATGCCTTG	TACGGGTCTT
Type IIb	Y1220_60	GGACAGACCA	TTGTTAATTG	TTGGTCTCGG	TCTTAACTAG	GAATGCCTTG	TACGGGTCTT
	GYB650_26	GGACAGACCA	TTGTTAATTG	TTGGTCTCGG	TCTTAACTAG	GAATGCCTTG	TACGGGTCTT

```
                    841                                                          900
Type I   GYB2180_57 TGGTTCAACA AACCACCCGG AATACGTCCC TGCCCTTTGT ACACACCGCC CGTCGCTCTT
         Y1211_4    TGGTTCAACA AACCACCCGG AATACGTCCC TGCCCTTTGT ACACACCGCC CGTCGCTCTT
Type IIa KH316_25   TGGTTCAACA AACCACCCGG AATACGTCCC TGCCCTTTGT ACACACCGCC CGTCGCTCTT
         GYA2395_38 TGGTTCAACA AACCACCCGG AATACGTCCC TGCCCTTTGT ACACACCGCC CGTCGCTCTT
Type IIb Y1220_60   TGGTTCAACA AACCACCCGG AATACGTCCC TGCCCTTTGT ACACACCGCC CGTCGCTCTT
         GYB650_26  TGGTTCAACA AACCACCCGG AATACGTCCC TGCCCTTTGT ACACACCGCC CGTCGCTCTT

                    901                                                          960
Type I   GYB2180_57 ACCGATGAAC TTCTTTGTGA GTCTAAGGGA CTGGGTTAAT ATTATTTT-- AATAGGTATA
         Y1211_4    ACCGATGAAC TTCTTTGTGA GTCTAAGGGA CTGGGTTAAT ATTATTTT-- AATAGGTATA
Type IIa KH316_25   ACCGATGAAC TTCTTTGTGA GTCTAAGGGA CTGGGTTAAT ACTATTTTAT AATAGTTATA
         GYA2395_38 ACCGATGAAC TTCTTTGTGA GTCTAAGGGA CTGGGTTAAT ACTATTTTAT AATAGTTATA
Type IIb Y1220_60   ACCGATGAAC TTCTTTGTGA GTCTAAGGGA CTGGGTTAAT ACTATTTT-- -ATAGTTATA
         GYB650_26  ACCGATGAAC TTCTTTGTGA GTCTAAGGGA CTGGGTTAAT ACTATTTT-- -ATAGTTATA

                    961                                                    1014
Type I   GYB2180_57 TACCTATGGA AACTTATACG AACAATGTGG TTTAAAGGAA AGAGAAGTCG TAAC
         Y1211_4    TACCTATGGA AACTTATACG AACAATGTGG TTTAAAGGAA AGAGAAGTCG TAAC
Type IIa KH316_25   TACCTATGGA AACTTATACG AACAATGTGG TTTAAAGGAA AGAGAAGTCG TAAC
         GYA2395_38 TACCTATGGA AACTTATACG AACAATGTGG TTTAAAGGAA AGAGAAGTCG TAAC
Type IIb Y1220_60   TACCTATGGA AACTTATACG AACAATGTGG TTTAAAGGAA AGAGAAGTCG TAAC
         GYB650_26  TACCTATGGA AACTTATACG AACAATGTGG TTTAAAGGAA AGAGAAGTCG TAAC
```
